# Neuroprotective and antioxidant properties of new quinolylnitrones in in vitro and in vivo cerebral ischemia models

**DOI:** 10.1038/s41598-023-29929-7

**Published:** 2023-02-17

**Authors:** Beatriz Chamorro, Sara Izquierdo-Bermejo, Julia Serrano, Dimitra Hadjipavlou-Litina, Mourad Chioua, Francisco López-Muñoz, José Marco-Contelles, Ricardo Martínez-Murillo, María Jesús Oset-Gasque

**Affiliations:** 1 Department of Biochemistry and Molecular Biology, Faculty of Pharmacy, Complutense University of Madrid, Plaza Ramón y Cajal s/n, Ciudad Universitaria, 28040 Madrid, Spain; 2 Faculty of Health, Camilo José Cela University, Villanueva de la Cañada, Madrid, Spain; 3 Neurovascular Research Group, Department of Translational Neurobiology, Cajal Institute (CSIC), Madrid, Spain; 4 Department of Pharmaceutical Chemistry, School of Pharmacy, Faculty of Health Sciences, Aristotle University of Thessaloniki, 54124 Thessaloniki, Greece; 5 Laboratory of Medicinal Chemistry, Institute of Organic Chemistry (CSIC), C/ Juan de la Cierva 3, 28006 Madrid, Spain; 6 Neuropsychopharmacology Unit, “Hospital 12 de Octubre” Research Institute, Madrid, Spain; 7 Center for Biomedical Network Research on Rare Diseases (CIBERER), CIBER, ISCIII, Madrid, Spain; 8 Instituto de Investigación en Neuroquímica, Universidad Complutense de Madrid, Ciudad Universitaria, 28040 Madrid, Spain

**Keywords:** Biochemistry, Chemical biology, Drug discovery, Neuroscience

## Abstract

Cerebral ischemia is a condition affecting an increasing number of people worldwide, and the main cause of disability. Current research focuses on the search for neuroprotective drugs for its treatment, based on the molecular targets involved in the ischemic cascade. Nitrones are potent antioxidant molecules that can reduce oxidative stress. Here we report the neuroprotective properties and the antioxidant power of the six new quinolylnitrones (**QNs**) **1**–**6** for their potential application in stroke therapy. **QNs 1**–**4** are 2-chloro-8-hydroxy-substituted **QNs** bearing *N*-t-butyl or *N*-benzyl substituents at the nitrone motif located at C3, whereas **QN5** and **QN6** are 8-hydroxy **QNs** bearing *N*-t-butyl or *N*-benzyl substituents at the nitrone motif located at C2, respectively. In vitro neuroprotection studies using **QNs 1–6** in an oxygen-glucose-deprivation model of cerebral ischemia, in human neuroblastoma cell cultures, indicate that all **QNs** have promising neuroprotective, anti-necrotic, anti-apoptotic, and anti-oxidant properties against experimental ischemia–reperfusion in neuronal cultures. **QN6** stands out as the most balanced nitrone out of all tested QNs, as it strongly prevents decreased neuronal metabolic activity (EC_50_ = 3.97 ± 0.78 μM), as well as necrotic (EC_50_ = 3.79 ± 0.83 μM) and apoptotic cell death (EC_50_ = 3.99 ± 0.21 μM). **QN6** showed high capacity to decrease superoxide production (EC_50_ = 3.94 ± 0.76 μM), similar to its parent molecule α-phenyl-tert-butyl nitrone (**PBN**) and the well-known anti-oxidant molecule *N*-acetyl-l-cysteine (**NAC**). Thus, **QN6** demonstrated the highest antioxidant power out of the other tested **QNs**. Finally, in vivo treatment with **QN6** in an experimental permanent stroke model elicited a significant reduction (75.21 ± 5.31%) of the volume size of brain lesion. Overall, **QN6** is a potential agent for the therapy of cerebral ischemia that should be further investigated.

## Introduction

Cerebrovascular disease is one of the leading causes of death and disability, with increasing prevalence due to the ageing of the population^1^. In the last decades, the major focus on our group has been the synthesis and pharmacological characterization of neuroprotective drugs for cerebral ischemia (CI)^2^, and Alzheimer's disease (AD)^3^ treatments, as these are highly impactful, devastating disorders that cause major socio-economic problems for societies and health-care systems worldwide.

CI is caused by the interruption of the blood supply to the brain. Most CIs (87%) are due to ischemic stroke, caused by thrombosis or embolisms^4^. The rest are due to hemorrhagic stroke, caused mainly by rupture of blood vessels or aneurysms. The interruption of the supply of oxygen and nutrients to the brain during CI causes damage to the brain tissue and the effects depend on which part of the brain is injured and how severely it is affected. Highly severe CI can cause sudden death, but stroke is more incapacitating than lethal, as it is the major cause of neurological disability and the second cause of dementia after AD.

Dementia is one of the most common ageing-associated disorders. In particular, vascular dementia (VD) refers to brain disorders where cognitive decline is of cardiovascular origin^5^. Nowadays, the term vascular cognitive impairment (VCI) is preferred as it reflects the full range of cognitive syndromes resulting from cerebrovascular disease, including the clinically well-defined CI and subclinical cerebrovascular injuries, with VD being the most severe expression. Cerebrovascular disease is also an adjuvant for the onset of dementia of variable origin, including AD and other neurodegenerative diseases. In fact, mixed forms of the former (VD/AD) are now believed to be much more common than the “pure” ones. Their pathogenic mechanisms and neuropathologies vary for each VCI subtype, which are issues that have not been sufficiently considered in this research area.

**Figure 1:**
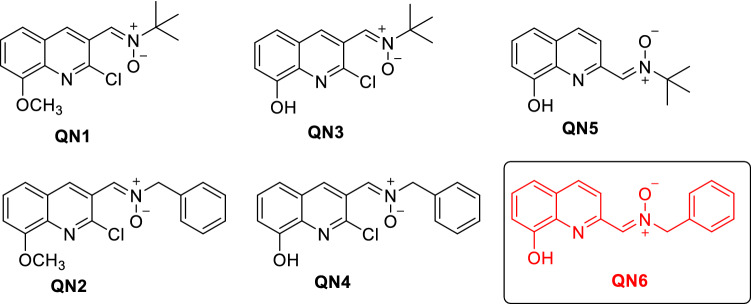
Structure of **QNs 1–6**.

In this context, we have recently designed and synthesized new quinolylnitrones (**QNs**) **1**–**6** (Fig. 1) as potential therapeutic antioxidants agents for VD^6^. **QNs 1**–**4** (Fig. 1) are 2-chloro-8-hydroxy substituted **QNs** bearing *N*-t-butyl or *N*-benzyl substituents at the nitrone motif located at C3, whereas **QN5** and **QN6** (Fig. 1) are 8-hydroxy **QNs** bearing *N*-t-butyl or *N*-benzyl substituents at the nitrone motif located at C2, respectively. We identified **QN6** (Fig. 1) as a potent inhibitor of human butyrylcholinesterase (hBChE) (IC_50_ = 1.06 ± 0.31 nM) and human monoamine oxidase B (hMAO-B) (IC_50_ = 4.46 ± 0.18 µM), which acts as a free radical-scavenger and biometal chelator that is permeable to the blood–brain-barrier (BBB). This compound was not cytotoxic and showed neuroprotective properties in the 6-hydroxydopamine-induced cell model of Parkinson’s disease^6^. Finally, in vivo studies demonstrated that **QN6** showed anti-amnesic effects in the scopolamine-induced mouse model of AD without adverse effects on motor function and coordination^6^. To sum up, these results allowed us to identify **QN6** as a promising candidate for the therapy of VD.

Given the tight link between CI and VD^7^, next we assessed whether **QNs 1–6** (Fig. 1) could be protective in the in vitro CI model where oxygen glucose deprivation (OGD) is followed by oxygen and glucose resupply (OGR) in human neuroblastoma cell cultures, this being an in vitro model of ischemia reperfusion (IR). In agreement with our previous AD findings, **QN6** prevented the decreased neuronal, metabolic activity, and increased necrotic and apoptotic cell deaths, which occur following IR. **QN6** also lowered superoxide production in human neuroblastoma cell cultures, showing overall the best profile out of all tested **QNs.** Finally, we carried out an in vivo*,* permanent, focal ischemia assay, where **QN6** remarkably reduced the brain infarcted volume after ischemia.

## Results and discussion

In this study a number of different experiments were carried out in order to characterize the neuroprotective and antioxidant properties of **QNs 1**–**6** (Fig. 1). These analyses were carried out in the SH-SY5Y human neuroblastoma cell line, one of the most commonly used cell lines in neuroscience as a screening model for studies of neuroprotection and neuroinflammatory protection^8^ that reveals oxidative stress-induced cell death^9^.

### Neuroprotection analysis

#### Basal neurotoxicity of **QNs 1–6, PBN** and **NAC**

**Figure 2:**
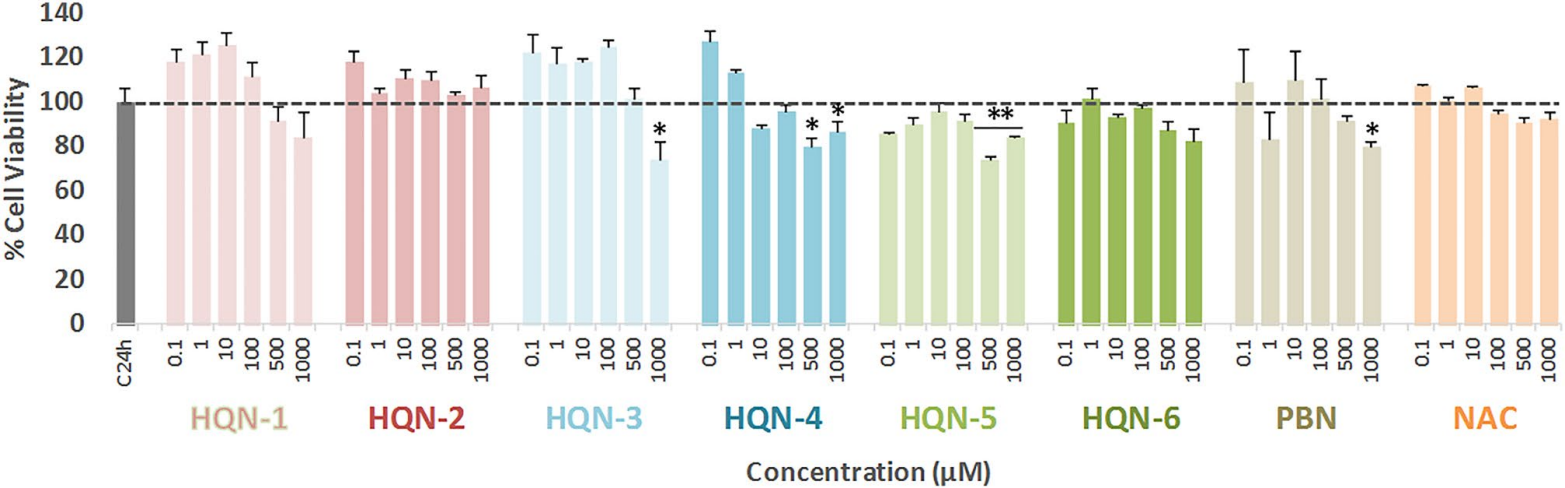
Effect of **QNs 1–6**, **PBN** and **NAC** on human neuroblastoma SH-SY5Y cell viability under basal conditions. Bars represent % of cell viability in the presence of the compounds at the indicated concentrations. Cell viability for the untreated cells (C24h) was assigned 100% (100 ± 5.52%, mean ± SEM). Values are the mean ± SEM of five experiments, each one performed in triplicate. Statistics was performed by one-way ANOVA test. There were no significant differences with respect to control. Analysis of results above 100% is not shown.

First, we assessed the neurotoxicity of **QNs 1–6** by measuring cell viability using an XTT assay in the presence of different compounds, but without adding any toxic insult. As shown in Fig. 2, **QNs 3**,** 4**,** 5** and **PBN** had a baseline neurotoxicity of 5–10% loss of viability at high concentrations (1000 μM), and **QNs 4** and **5**, at the concentration of 500 μM. This small neurotoxic effect was taken into account when calculating neuroprotection.

#### Neuroprotection analysis in an oxygen and glucose deprivation (OGD) followed by oxygen and glucose resupply (IR) model

The neuroprotective effect of **QNs 1–6**, α-phenyl-tert-butyl nitrone (**PBN**) and *N*-acetyl-l-cysteine (**NAC**), used as standards, was evaluated in an in vitro oxygen glucose deprivation (OGD) model, followed by oxygen and glucose resupply (OGR), an in vitro model of ischemia reperfusion (IR)^10^.

**Figure 3:**
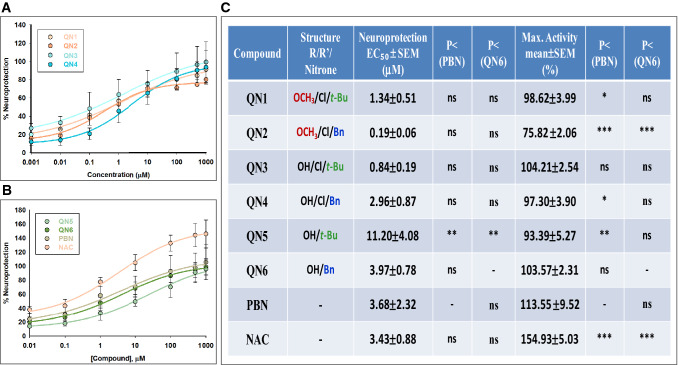
Neuroprotective effects of **QNs 1–6**, **PBN** and **NAC** against lost of cell metabolic capacity induced by IR treatment in SH-SY5Y human neuroblastoma cells. (**A**,**B**) Dose–response curves showing the % neuroprotection of **QNs 1**–**4** (**A**) and **QNs 5**–**6**, **PBN** and **NAC** (**B**) at the indicated concentrations. The curve adjustments to estimate the EC_50_ and MNA were carried out by non-linear ponderated regression analysis of minimal squared, using logistic curves f1 = min + (max − min)/(1 + (x/EC_50_)^(− Hillslope). Data represent mean ± SEM of four experiments, each one performed in triplicate. The analysis was implemented using the software SigmaPlot v.11 (Systat Software INC., 2012). (**C**) EC_50_ and MNA values for the indicated compounds. The statistics compares the differences between EC_50_ or MNA values for different compounds tested against **PBN**, or **QN6** at *P < 0.05, **P < 0.01, ***P < 0.001 (ANOVA one way).

Based on our previous experience^10^, the concentrations of the tested compounds ranged from 0.01 to 1000 μM, after IR. To measure cell viability, XTT test was used to evaluate cellular metabolic capacity^8,9^. After OGD (I) (4 h), a loss of metabolic activity of about 45% (45.22 ± 2.88% cell viability, mean ± SEM; n = 6) was observed, showing a small cell recovery after 24 h reperfusion (IR) of about a 15% (62.28 ± 6.19% cell viability; mean ± SEM; n = 6) (Fig. S1, Supplementary Material). As shown in Fig. S1, all **QNs** were highly effective as neuroprotective agents, reversing the IR-induced decrease cell viability (loss of metabolic activity) in a concentration-dependent manner, and with similar potency to **PBN** and **NAC**. To get a clearer picture, and be able to establish the differences between them, we carried out the effective dose 50 (EC_50_) and maximal neuroprotective activity (MNA) analyses. The analyses of concentration–response curves for **QNs 1–6**, compared with **PBN** and **NAC**, in the range of 0.01 μM to 1 mM, are presented (Fig. 3A,B), showing the corresponding EC_50_ values and the highest neuroprotective activities (Fig. 3C). As shown in Fig. 3C, the EC_50_ values, from the lowest to the highest, i.e. from more to less neuroprotective, follows the order: **QN2 ≤ QN3 ≤ QN1 ≤ QN4 ≤ NAC ≤ PBN ≤ QN6 < QN5**. These results indicate that only the EC_50_ for **QN5** was significantly higher than that of the other nitrones and **NAC**. On the other hand, the comparison of the MNA shows the following order of potency: **NAC >> PBN ≥ QN3 ≥ QN6 > QN1 ≥ QN4 ≥ QN5 ≥ QN5 > QN2**, indicating that the least effective nitrones were **QN5** and **QN2**, which had a significantly lower MNA than the rest of the other nitrones and **NAC**.

All **QNs**, with the exception of **QN5**, have similar EC_50_ values, and not significantly different from **PBN** and **NAC**, although the MNA of **QNs 1**,**2**,**4** was lower than that of the reference compounds, leaving **QN3** and **QN6** as the most balanced nitrones in terms of the neuroprotective capacity against ischemia-induced loss of cellular metabolic capacity. In terms of the structure–activity relationship (SAR), although the presence of the chloro and methoxy substituents at the QN core slightly reduces the EC_50_ values, it also reduces MNA, so they do not globally produce greater neuroprotection. Regarding the presence of the Bn or *t*-Bu substituents, no significant different neuroprotective activity was observed among the nitrones bearing them, with the exception of **QNs** without Cl, such as **QN5** and **QN6**, where the Bn substituent seems to confer lower EC_50_ and higher neuroprotective MNA.

In conclusion, all **QNs** have potent neuroprotective capacities in terms of recovery of the overall metabolic activity. However, the two most balanced nitrones in terms of both affinity and efficacy were **QN3 and QN6**, the latter showing a low EC_50_ of 3.97 ± 0.78, similar to that of **PBN** and **NAC**, and a high MNA of 113.57 ± 10.31%, similar to that of PBN. For this reason, and based on the results on cell death and antioxidant capacity (see below), we decided to perform statistical comparisons against this compound (Fig. 3C).

#### Effect of **QNs 1–6** on necrotic cell death induced by oxygen and glucose deprivation followed by oxygen and glucose resupply (IR) model

In order to further investigate the neuroprotective effect of these **QNs**, next we studied their neuroprotective effect on necrotic and apoptotic cell death, two types of cell death that occur during cerebral ischemia. During an ischemic stroke, there is massive cell death due to necrosis, and, as a consequence, the plasma membrane is broken or significantly permeabilized. Under these circumstances, lactate dehydrogenase (LDH), a soluble cytosolic enzyme, easily crosses the damaged membrane, and for this reason, it is possible to determine the extent of the cell necrosis taking place in the OGD experiment by comparing its extracellular to its intracellular activity^11^.

In Fig. S2 (Supplementary Material) we have gathered the values obtained from the measurement of LDH release after OGD for 4 h, followed by 24 h reperfusion (IR) on neuroblastoma cells, by adding nitrones **QNs 1–6** at 0.1–1000 μM concentrations, and **PBN** and **NAC.** Thus, we conclude that all the **QNs** significantly decreased the release of LDH in a concentration-dependent manner, reaching a 100% of maximal inhibition of LDH release at concentrations between 100 and 1000 μM (Fig. S2, Supplementary Material).

**Figure 4:**
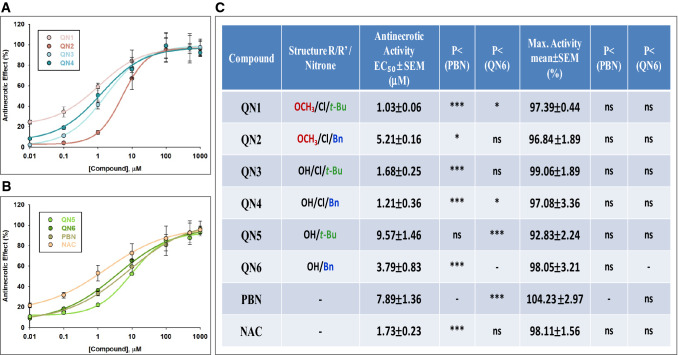
Anti-necrotic effects of **QNs 1**–**6**, **PBN** and **NAC** against necrotic cell death induced by IR treatment in SH-SY5Y human neuroblastoma cells. (**A**,**B**) Dose–response curves showing the % of anti-necrotic effect of different compounds: **QNs 1**–**4** (**A**) and **QNs 5**, **6**, **PBN** and **NAC** (**B**) at the indicated concentrations. The curve adjustments to estimate the EC_50_ and MNA were carried out by non-linear ponderated regression analysis of minimal squared, using logistic curves f1 = min + (max − min)/(1 + (x/EC_50_)^(− Hillslope). Data represent mean ± SEM of four experiments, each one performed in triplicate (n = 4). The analysis was implemented using the software SigmaPlot v.11 *(Systat Software INC., 2012)*. (**C**) EC_50_ and MNA values for the indicated compounds. The statistics compares the differences between EC_50_ or MNA values for different compounds tested against **PBN**, or **QN1** at *P < 0.05, **P < 0.01, ***P < 0.001 (ANOVA one way).

In order to compare the efficacy of these nitrones in abolishing necrotic cell death, we performed a dose–response study by determining the EC_50_ and the MNA. Thus, in Fig. 4 we have gathered the analysis of the concentration–response curves for **QNs 1–6,** compared with **PBN** and **NAC**, in the range of 0.01 μM to 1 mM (Fig. 4A,B) showing the corresponding EC_50_ values and the highest anti-necrotic activities (Fig. 4C).

As shown in Fig. 4C, the EC_50_ values, from the lowest to the highest, i.e. from more to less anti-necrotic compounds, follows the order: **QN1 ≤ QN4 < QN3 ≤ NAC ≤ QN6 ≤ QN2 ≤ PBN ≤ QN5**. By contrast, there were not statistic significant differences between the MNA of the different nitrones, values which showed the following order of potency: **PBN ≥ QN3 ≥ NAC ≥ QN6 ≥ QN1 ≥ QN4 ≥ QN2 ≥ QN5**. In other words, the nitrones with the highest anti-necrotic efficacy were **QN1, QN3** and **QN4**, as they had the lowest EC_50_, lower than **PBN** and similar to **NAC**, and the same MNA as these and the other nitrones. As for the anti-necrotic capacity of **QN6**, although it has a slightly worse EC_50_, which is significantly higher than **QN1**, **QN4** and **NAC**, **QN6** showed one of the best maximal anti-necrotic activities (Fig. 4C). Therefore, **QN6** was shown to be a balanced nitrone in terms of its anti-necrotic capacity.

Taking all of these results together, and from the SAR point of view, we found the following differences when comparing with the results of metabolic capacity of the **QNs** (cell viability (measured with XTT): (1) The **QNs** with the highest anti-necrotic capacity (EC_50_) are those containing the chloro and methoxy substituents in their structure, with the exception of **QN2**, which had the highest EC_50_ and one of the lowest MNA. **QNs 5** and **6** bearing no chloro atom in their structure show lower anti-necrotic effects than the rest of the tested compounds, **QN5** had the highest EC_50_ and the lowest MNA, and, therefore, is the worst nitrone, whereas **QN6** is a balanced nitrone in terms of both parameters, as shown in the viability studies with XTT. (2) As in the case of the cell viability study, the Bn or t-Bu substituents do not affect significantly the neuroprotective activity of the nitrones, although in the group of **QNs** bearing no chloro atom in their structure, such as **QNs 5** and **6**, the Bn substituent affords more anti-necrotic potential, in a similar way to the cell viability experiments (lower EC_50_ and higher MNA of **QN6** vs **QN5**).

To sum up, all the **QNs** have potent anti-necrotic activity, **QNs 1**, **3**, **4** and **6** being the most powerful and balanced, showing an anti-necrotic power similar to **NAC** and better than **PBN**.

#### Effect of **QNs 1–6** on apoptotic cell death induced by oxygen and glucose deprivation followed by oxygen and glucose resupply (IR) model

Next, and in order to evaluate the extent of cell death by apoptosis, we determined the caspase-3 activity, by using DEVD-AMC as a substrate, which affords fluorescent AMC upon hydrolysis. So, after OGD (4 h), and adding nitrones **QNs 1–6**, **PBN** and **NAC**, at 0.1–1000 μM concentrations, followed by IR (24 h), the cells were lysated, DEVD-AMC was added, and the fluorescence measured.

As shown in Fig. S3 (Supplementary Material), in general, all the tested **QNs** had a concentration-dependent anti-apoptotic effect. However, the anti-apoptotic effect was achieved at higher concentrations than the anti-necrotic effect. Moreover, in nitrones bearing no chloro atom in their structure (**QNs 5** and **6**), the anti-apoptotic effect is better than their anti-necrotic effect.

**Figure 5:**
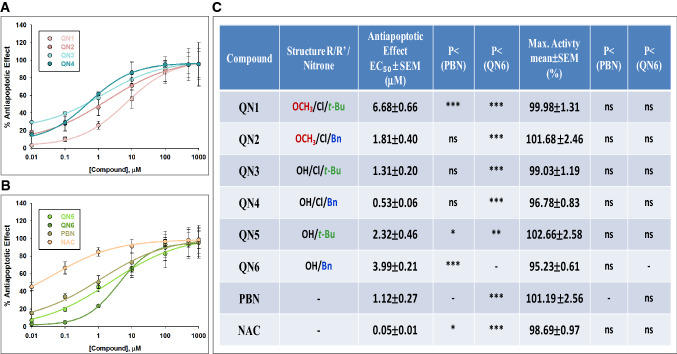
Neuroprotective effects of **QNs 1**–**6**, **PBN** and **NAC** against apoptotic cell death induced by IR treatment in SH-SY5Y human neuroblastoma cells. (**A**,**B**) Dose–response curves showing the % neuroprotection of different compounds: **QNs 1**–**4** (**A**) and nitrones **QNs 5**–**6**, **PBN** and **NAC** (**B**) at the indicated concentrations. The curve adjustments to estimate the EC_50_ and MNA were carried out by non-linear ponderated regression analysis of minimal squared, using logistic curves f1 = min + (max − min)/(1 + (x/EC_50_)^(− Hillslope). Data represent mean ± SEM of three experiments, each one performed in triplicate. The analysis was implemented using the software SigmaPlot v.11 (Systat Software INC., 2012). (**C**) EC_50_ and MNA values for the indicated compounds. The statistics compares the differences between EC_50_ or MNA values for different compounds tested against **PBN**, or **QN4** at *P < 0.05, **P < 0.01, ***P < 0.001 (ANOVA one way).

In order to compare the order of efficacy of these nitrones in abolishing apoptotic cell death, we performed a dose–response study by determining the EC_50_ and the MNA. Thus, Fig. 5 gathers the analyses of concentration–response curves for **QNs 1**–**6**, compared to **PBN** and **NAC**, in the range of 0.01 μM to 1 mM (Fig. 5A,B) showing the corresponding EC_50_ values and the highest, anti-apoptotic activities (Fig. 5C).

As shown in Fig. 5C, the EC_50_ values, from the lowest to the highest, i.e. from more to less anti-apoptotic compounds, follows the order: **NAC <<< QN4 ≤ PBN ≤ QN3 ≤ QN2 ≤ QN5 ≤ QN6 ≤ QN1**. By contrast, there were not statistically significant MNA differences among the tested nitrones, showing values with the following order of potency: **QN5 ≥ PBN ≥ QN2 ≥ QN1 ≥ QN3 ≥ NAC ≥ QN4 ≥ QN6**. In other words, all the nitrones had a very high MNA, **QNs 4**, **3** and** 2** having the highest anti-apoptotic potency, similar to **PBN**, although lower than **NAC**, which showed the greatest anti-apoptotic power.

Considering all these results together, and from the SAR point of view, we found the following differences between the anti-necrotic and metabolic capacities for the tested QNs: (1) The anti-necrotic and anti-apoptotic effects of the different **QNs** influence the general metabolic state of the cell in different ways. Thus, the strongest anti-necrotic **QNs** (lower EC_50_ and/or higher Maximal Activity)**,** namely **QNs 1**, **3**, **4** and **6**, also proved the most efficient at reversing the metabolic alterations, and were therefore considered as the most neuroprotective nitrones. In contrast, the **QNs** with poor anti-necrotic activity (higher EC_50_ and/or lower Maximal Activity), namely **QNs 2** and **5**, were showed to be the least neuroprotective compounds. This conclusion is further confirmed by the fact that **QNs 2** and **5** have a very good anti-apoptotic activity, but this capacity is not reflected in the overall neuroprotective effect. So, the improvement in overall cell viability induced by **QNs** depends more on their anti-necrotic effect than on their anti-apoptotic effect. (2) Similar to the cell viability and anti-necrotic assessments, the Bn or t-Bu substituents significantly affected the anti-apoptotic activity of these **QNs**. However, among **QNs** with chloro in their structure, the Bn substituent, and not t-Bu, provides more anti-apoptotic potential. Moreover, for **QNs 5** and **6** bearing no chloro atom, in opposite trend as shown in the cell viability and anti-necrotic experiments, it is the t-Bu, but not the Bn substituent that confers lower EC_50_ and higher MNA.

To sum up, all **QNs** have very good anti-apoptotic activity, **QNs 2**,** 4** and **5** being the most powerful and balanced, with an anti-apoptotic power similar than **PBN**, although lower than **NAC**. Thus, the anti-necrotic or anti-apoptotic effect of these compounds seems to condition their effect on overall cellular metabolic activity.

### Antioxidant assays

#### Antioxidant capacity of** QNs 1–6**, **PBN** and **NAC**: production and scavenging of superoxide radical in human neuroblastoma SH-SY5Y cells

The results shown in the previous sections prompted us to investigate whether the observed neuroprotection was a consequence of their capacity to act as antioxidants and ROS scavengers, particularly of superoxide radical anion (O_2_^**·**−^). O_2_^**·**−^ detection was carried out by using dihydroethidium (DHE), after OGD (3 h) and IR (3 h), with or without **QNs 1–6**, including **PBN** and **NAC**, using compound concentrations from 0.1 to 1000 μM, after IR.

As shown in Fig. S4 (Supplementary Material), ROS level production after IR (0.277 ± 0.009 UAF/min/100,000 cells, 100 ± 3.47% of ROS release; means ± SEM; n = 6) was lower, but non-significantly different (ns, one way Anova test) than ROS production under OGD alone (0.297 ± 0.008 UAF/min/100,000 cells, 107.55 ± 4.35% of ROS release (means ± SEM; n = 6). As expected, **QNs 1**–**6** were able to partially or totally reverse the increase in ROS levels induced by IR, in a concentration-dependent manner (Fig. S4, Supplementary Material).

**Figure 6:**
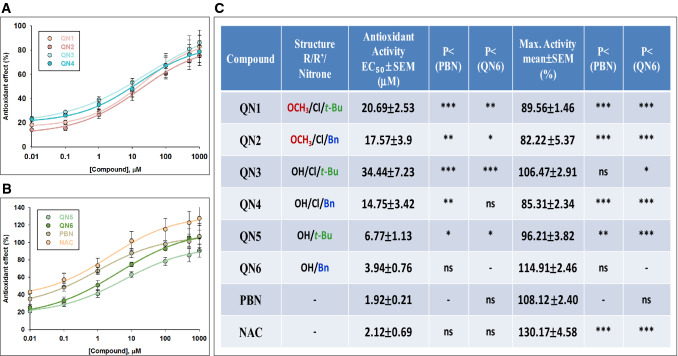
Antioxidant effect of **QNs 1**–**6**, **PBN** and **NAC** after I/R in human neuroblastoma SH-SY5Y cells. (**A**,**B**) Dose–response curves showing the percentage of antioxidant effect of different compounds at the indicated concentrations. The curve adjustments to estimate the EC_50_ were carried out by non-linear ponderated regression analysis of minimal squared, using logistic curves f1 = min + (max − min)/(1 + (x/EC_50_)^(− Hillslope). Data represent mean ± SEM of three experiments, each one performed in triplicate (n = 3). The analysis was implemented using the software SigmaPlot v.11 (Systat Software INC., 2012). (**C**) EC_50_ and maximal antioxidant activities (MAA) values for the indicated compounds. The statistics compares the differences between EC_50_ or MAA values for different compounds tested against **PBN** or **QN6** at *P < 0.05, **P < 0.01, ***P < 0.001 (one-way ANOVA, followed by Holm–Sidak analysis as a post hoc test).

The analyses of concentration–response curve data (Fig. 6A,B) and calculations of EC_50_ and the maximal antioxidant activities (MAA) of **QNs 1–6**, **PBN** and **NAC** are shown in Fig. 6C. The EC_50_ values from the lowest to the highest, i.e. from more to less antioxidant potency, follow the order: **PBN ≤ NAC ≤ QN6 < QN5 < QN4 ≤ QN2 ≤ QN1 < QN3**. There were also significant differences in the MAA of the different nitrones, values which showed the following order of potency: **NAC ≥ QN6 ≥ PBN ≥ QN3 > QN5 > QN1 ≥ QN4 ≥ QN2**. Thus, **QNs** differ greatly between them in their antioxidant capacity, both in EC_50_ and MAA. In this case, and in contrast to the previous assays, the most potent antioxidants were **QNs 6** and **5** bearing no chloro atom^−^ in their structure. In particular, **QN6** showed the highest antioxidant potency, in the same order that **PBN** and **NAC**, as reflected in EC_50_ and MAA.

Taking into account the SAR point of view, we can conclude that the antioxidant capacity is strongly related to the structure of the **QNs**, with the most potent being those without chloro atom in their structure, followed by those containing hydroxy and chloro substituents, and the weakest antioxidants being those with methoxy and chloro substituents in their structure. In addition, and regarding the influence of the t-Bu or Bn substituents, it can be clearly seen that the Bn group provides greater antioxidant capacity in all the compounds tested.

In conclusion, unlike its anti-necrotic and anti-apoptotic activities, the MAA is possessed by nitrones bearing no chloro atom in their structure, especially **QN6**, which places this nitrone as the most balanced in all the activities tested, with a good neuroprotective capacity at both the metabolic, anti-necrotic and antioxidant power by scavenging O_2_^**·**−^ in human neuroblastoma SH-SY5Y cells.

#### Antioxidant in vitro tests

The antioxidant capacity and the multifunctional biological properties of nitrones as possible therapeutic agents for vascular dementia and cerebral ischemia are known^12^. Thus, we decided to evaluate **QNs 1**–**6** in other antioxidant assays and to test them as possible anti-inflammatory agents examining their ability to inhibit soybean LOX. Four different antioxidant assays were used in order to obtain reproducible results, since factors like solubility or steric hindrance, which may be of overriding importance in one environment but not in another, can influence the antioxidant ability of a compound studied in a variety of experimental conditions. Thus, nitrones **QNs 1**–**6** were studied for their ability to compete DMSO for hydroxyl radicals, in the decolorizing 2,2′-azino-bis (3-ethylbenzthiazoline-6-sulfonic acid) (ABTS) test, to inhibit the lipid peroxidation (ILPO) and lipoxygenase (LOX). **PBN**, nordihydroguaiaretic acid (**NDGA**) and **Trolox** were used as standards for comparative purposes.

As shown in Table 1, **QN5** highly inhibits soybean **LOX** (IC_50_ = 5 µM, 11.1-fold less active than standard **NDGA**), followed by **QN4**. No inhibition was observed by **QN2** and **QN3**. **QN6** exhibits low inhibition. **QNs 3**–**5** significant inhibit lipid peroxidation, the lipophilicity playing an important role, as higher values lead to lower inhibition e.g., nitrones **QN2** and **QN6**, whereas **QNs 3**–**5** present a mean lipophilicity value of 2.39 which is close to the 2 ± 0.5 value for brain penetration. **QN5**, especially presents the optimum logP value 2.49, and is the most potent (81.5%), in the same range as standard **Trolox** (93%).

**Table 1:** In vitro antioxidant activity of nitrones **QNs 1**–**6**^a^.

Standards/nitrones	Clog *P*	LOX (%)/IC_50_	ILPO (%)	^.^OH (%)	ABTS^+^ (%)
NDGA	nd	0.45 μΜ	nd	nd	nd
Trolox	nd	nd	93	73	91
PBN	3.02	23%	11	na	5
QN1	nd	nd	nd	nd	nd
QN2	2.74	na	32	71	20
QN3	2.08	na	75	90	65
QN4	2.61	52.5 μΜ	60	na	77.4
QN5	2.49	5 μΜ	81.5	83.3	8
QN6	3.03	26%	45	22	81.4

^a^Nitrones tested at 100 µM. Values are means ± SD of three or four different determinations. Means within each column differ significantly (p < 0.05). *nd* not determined, *na* no activity.

**QN2**, **QN3** and **QN5** highly compete with DMSO for hydroxy radicals, the most potent being **QN3** (90%) followed by **QN5** (83.3%), both more potent than standard **Trolox** (73%). **QN3**, **QN4** scavenge potently the cationic radical ABTS^**·**+^ with **QN6** presenting the highest ability (81.4%), in the same range that standard **Trolox** (91%).

To sum up, **QN5** presents a combination of significant antioxidant activities being a potent anti-LOX agent. The antioxidant profile is correlated with the inhibitory activity since LOX inhibitors are acting as antioxidants. The quinoline nitrone group with the hydroxy substitution and the presence of the t-butyl group describes structurally a potent lead compound, whereas the presence of a chlorine does not seem to guarantee LOX inhibition, as in the structure of the most potent nitrone **QN5** no chlorine atom exists as a substituent.

However, and considering the overall biological behavior, as described above, we decided to selected **QN6**, the most potent ABTS^•+^ scavenging agent (Table 1), to study its neuroprotection in an in vivo pMCAO stroke model. The ABTS method is rapid, technically simple, and can be used over a wide range of pH values, in both aqueous and organic solvent systems, which enables the antioxidant capacity of both hydrophilic and lipophilic compounds to be determined with the same basic methodology; in addition, it also has good repeatability and is simple to perform; hence, it is widely reported^13^. Furthermore, the fact that the ABTS method, however, has not been correlated with biological effects, and consequently, its actual relevance to in vivo antioxidant efficacy is unknown, was an additional point of interest and challenge to test **QN6** in the in vivo stroke model.

#### Contribution of **QN6** to brain damage prevention

To corroborate the neuroprotective effect of **QN6** on in cell-based ischemia models, we induced a permanent middle cerebral artery occlusion (pMCAO) and evaluated infarct size at 48 h of reperfusion after pMCAO in mice treated with an intraperitoneal injection of **QN6**. As indicated by the Stroke Academic Industry Roundtable (STAIR), permanent ischemia animal models reflect the most frequent variants of stroke in patients who are outside of therapeutic windows, non-responders to r-tPA or surgical thrombectomy. In addition, permanent ischemia (no reperfusion) has also been associated with substantial accumulation of free radical reactive nitrogen and oxygen species^14^. pMCAO carried out in this study is a commonly used stroke in vivo model in mice^15^. Using the pMCAO procedure, we analyzed by Magnetic Resonance Image (MRI) the in vivo contribution of **QN6** to brain damage prevention by determining the resulting infarct volume at 48 h after surgery.

**Figure 7:**
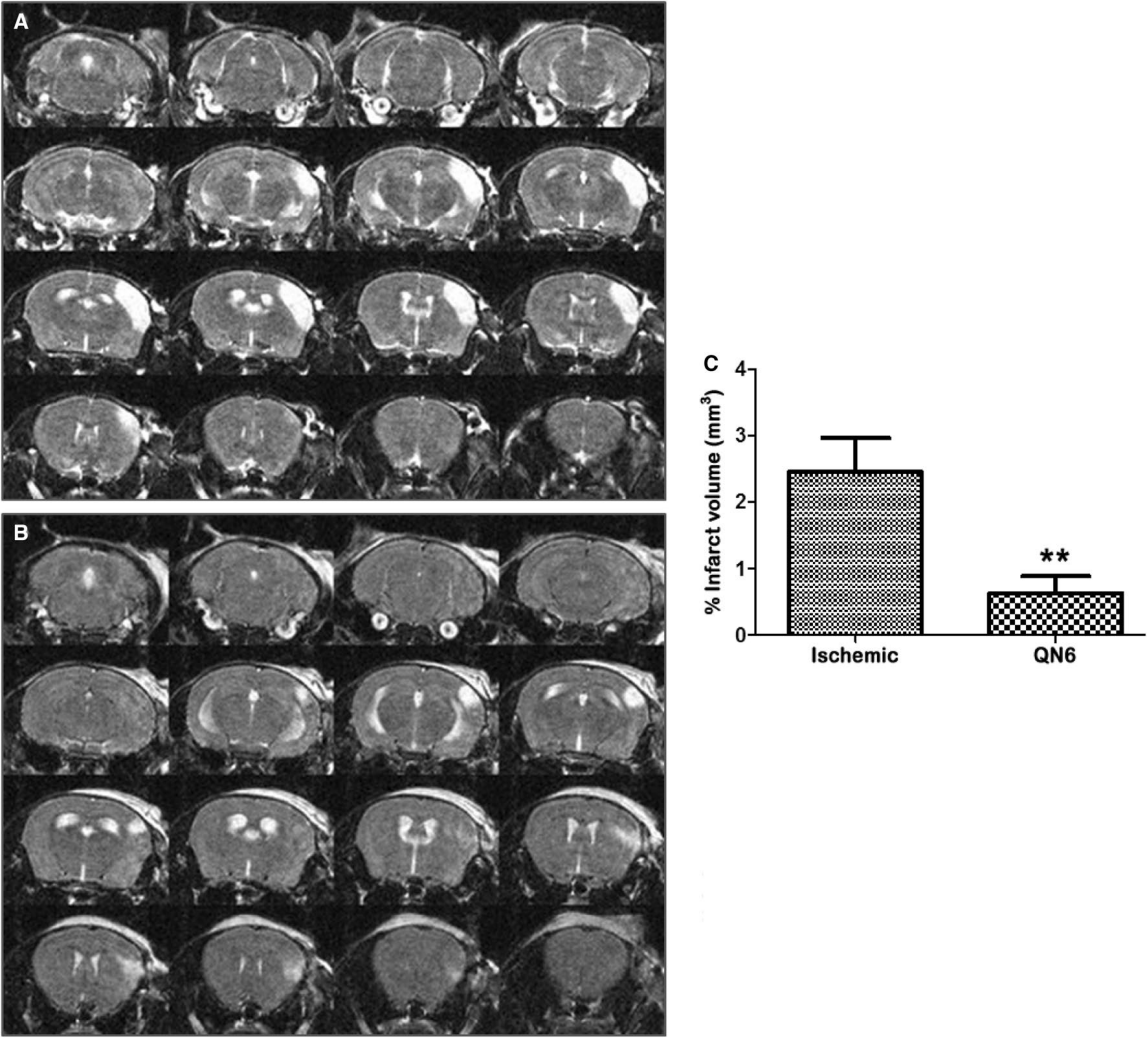
MRI study of **QN6** after 48 h of surgery. Note the lesion with T2WIhyperintensity restricted to the left side of the cerebral cortex. Treatment with **QN6** reduces the infarct volume after pMCAO in mice. Studies were performed 48 h after pMCAO. T2WI taken from vehicle **b** treated group (**A**) and **QN6 c** treated group (**B**) randomly chosen ischemic mice are shown. Infarct volumes were quantified from T2WI using ImageJ free software (**C**). Graphic represents the percentage of the infarct volume with respect to the total volume of the brain. Data are mean ± SEM, n = 7–8. Statistical significance was evaluated by two-way ANOVA followed by post hoc Bonferroni’s test to compare paired experimental points (**p < 0.01).

According to the experimental methods, the experiments compared infarct volume outcome between group’s b and c (Fig. 7A,B). Sham operated control group (a) showed with certainty that stroke was not due to the surgical pre-occlusive procedure. Infarct volumes, shown in mm^3^ were obtained integrating infarcted areas by counting pixels contained within the regions of interest. Each side of the coronal sections was sampled. Compared with vehicle-treated animals (b), a significant reduction in the percentage of brain infarct volume (75.21 ± 5.31%; P < 0.01) was appreciated in **QN6** treated group (c) (Fig. 7C). With regard to the whole brain volume, the infarct volume in group (b) represented 2.453%, whereas in group (c) dropped to 0.6262%. Statistical significance was evaluated by two-way ANOVA followed by post hoc Bonferroni’s test Student to determine the statistical significance of differences of infarct values between vehicle (b) and the **QN6** treated (c) mice. P value < 0.05 was considered significant.

## Conclusions

In this work, we report the neuroprotection profile of six **QNs 1–6** by assessing their effects on neuronal metabolic activity, necrotic and apoptotic cell death, as well as their antioxidant activity against ROS production**.**

**QNs** were shown to be potent neuroprotective, anti-necrotic, anti-apoptotic and anti-oxidant agents against ischemia–reperfusion-induced cell damage in neuronal cultures. **QN6** stood out as the most balanced molecule in terms of its neuroprotective, anti-necrotic and anti-oxidant capacity, although other nitrones showed mildly higher anti-apoptotic capacity than **QN6**.

The anti-necrotic and anti-apoptotic properties of **QNs** were opposite in the different **QNs**. **QNs 1**, **3**, **4**, and **6** showed better anti-necrotic than anti-apoptotic properties, while **QNs 2** and **5** afforded stronger anti-apoptotic than anti-necrotic protection. Anti-necrotic activity correlated more strongly with global neuroprotection than the anti-apoptotic capacity did.

Regarding the structure–activity relationship among **QNs**, the presence of Cl or the Bn or t-Bu substituents, did not significantly affect their neuroprotective, anti-necrotic or anti-apoptotic effects, although they affected their anti-oxidant capacity. Thus, their anti-oxidant activity is strongly related to the structure of **QNs**, with the most potent being those without chloro atom in their structure (**QN6** and **QN5**). In addition, the Bn group provides greater anti-oxidant capacity in all the compounds tested. Therefore, **QN6** was the best nitrone for its ability to lower superoxide levels in neuronal cultures and for its ability to scavenge the cationic radical ABTS^•+^ in in vitro tests, while **QN5** was better for its capacity to inhibit ILPO and LOX activities in in vitro tests.

**Figure 8:**
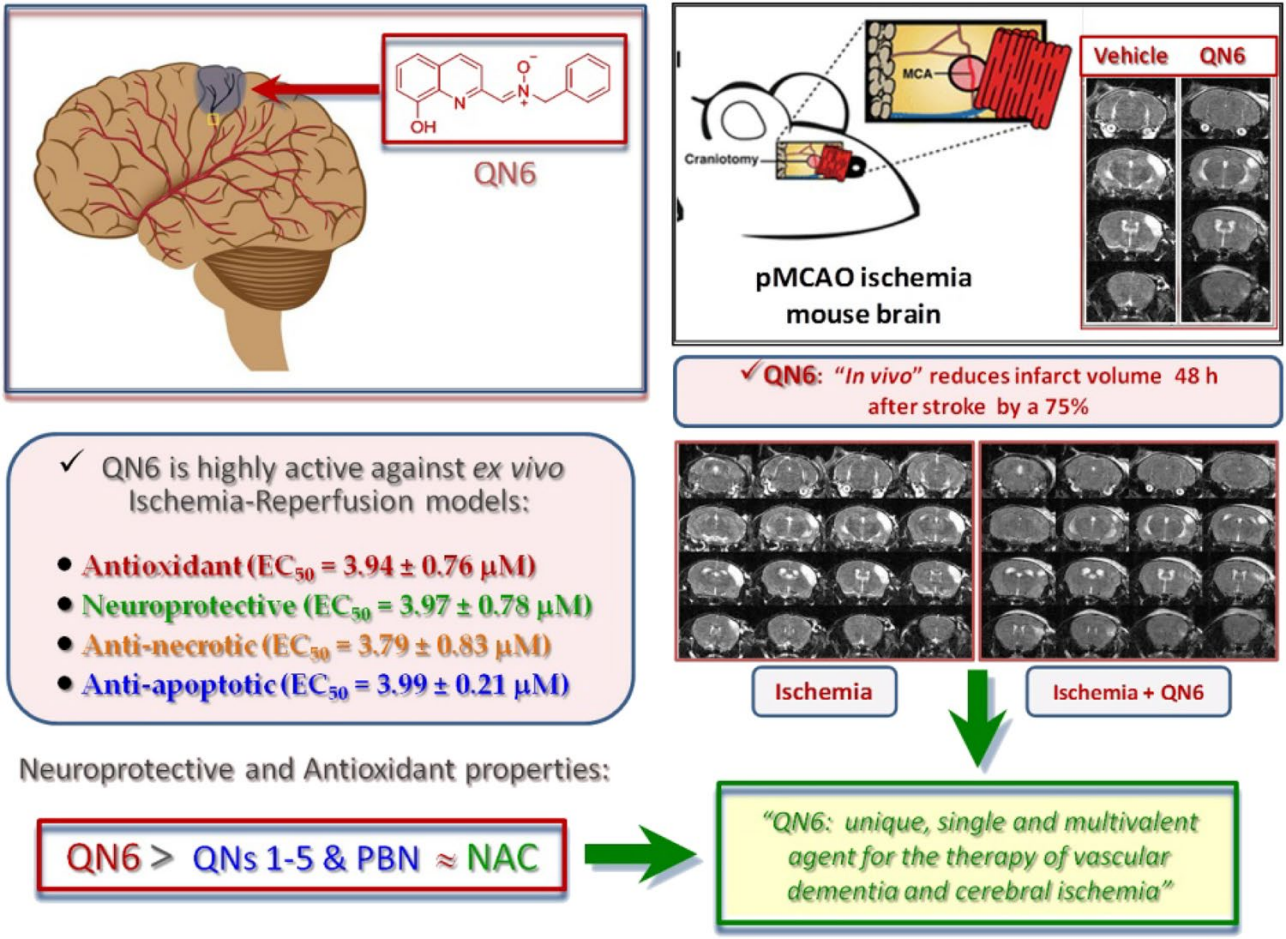
**QN6** as a single multivalent agent for the therapy of vascular dementia and cerebral ischemia.

Finally, regarding the ability of **QN6** to decrease cerebral infarct size in in vivo experiments, this molecule caused a strong reduction (70–75%) of the pMCAO-induced average size of the infarcted areas (c), compared to the vehicle treated group (b). In sum, the results of this novel study support the in vivo ability of **QN6** to penetrate through the BBB, and its potential as a new promising therapeutic agent for the therapy of stroke (Fig. 8). Additionally, the strong capacity of **QN6** to trap ABTS^•+^ *vs* other antioxidants tests, may be used as a diagnostic tool to select anti-oxidant agents for in vivo tests and pre-clinical assays (Fig. 8).

## Methods

### Chemistry

**QNs 1**–**6** were prepared following methods previously described in our laboratory, and were identified and characterized using the appropriate analytical and spectroscopic data^6^.

### Neuroprotection in vitro assays

#### Neuroblastoma cell cultures

SH-SY5Y human neuroblastoma cell line cultures were carried out in flasks containing Dulbecco’s: Ham’s F12, 1:1 [v/v] containing 0.5 mM sodium pyruvate, 2.5 mM GlutaMAX™ (GIBCO, Life Technologies, Madrid (Spain), 3.15 mg/mL glucose, 1% Antibiotic–Antimycotic (100 ui/mL penicillin, 100 mg/mL streptomycin, and 0.25 mg amphotericin B), 1% Gentamicin 15 mg/mL (Sigma-Aldrich, Madrid, Spain) and 10% Fetal Calf Serum (FCS) (Gibco; Life Technologies, Madrid, Spain) as described^10^. For each experiment, neuroblastoma cells were digested partially with 3 mL of 0.25% trypsin–EDTA and detached cells were seeded in plates of 48 or 96 wells at a density of 0.5 or 1.25–1.5 × 10^5^ cells/well, respectively, depending on the assay. When the seeded cells reached 80% confluence, renewal of the medium was performed and the pertinent assays were carried out.

#### Neuroblastoma cell cultures exposure to oxygen–glucose deprivation (OGD)

For the induction of experimental ischemia, and its sub-consequent cell damage, the neuroblastoma cells cultured were exposed to OGD for 3–4 h in a glucose-free Dulbecco’s medium inside an anaerobic chamber in the presence of a gas mixture of 95% N_2_/5% CO_2_ and humidified at 37 °C at a constant pressure of 0.15 bar. After the OGD period, medium was replaced by oxygenated medium containing 10% FCS, and cells were maintained in normoxic conditions for 24 h to recovery (R24h). The molecules used in the neuroprotection studies were quinolylnitrones **QNs 1–6**, α-phenyl-*N*-tert-butyl nitrone (**PBN**) and *N*-acetyl-l-cysteine (**NAC**) (0.01–1000 µM), were added at the beginning of R24h as described^8^. In parallel, controls were performed by preserving these cells in Dulbecco’s medium containing glucose in an incubator in conditions of normoxia during the OGD period (3‒4 h); afterwards, the medium was changed, and the neuroblastoma cells (in the presence or the absence of the different molecules under study) were returned to the incubator for 24 h of recovery, as described^8^. Also, experiments including the same amounts of vehicle (final concentration < 1% dimethyl sulfoxide) were performed, serving as controls. The effects of **QNs 1**–**6** were analyzed three-five times independently using different culture batches, and each experiment was carried out in triplicate.

#### Evaluation of cell viability

Measurement of cell viability was carried out in SH-SY5Y human neuroblastoma cells using 96-well culture plates, containing about 0.5 × 10^5^ cells/well, after the OGD-R24h or control treatment including 0.01–1000 µM of the different testing compounds (**QNs 1**–**6**, **PBN**, and **NAC**), using the Cell Proliferation Kit II (XTT) (Sigma Aldrich, Madrid, Spain), which evaluates cell’s metabolic capacity, as described by Xie et al. and Yang et al.^8,9^. XTT solution (final concentration 0.3 mg/mL) was added to the culture cells, incubated for 2 h at 37 °C with 5% CO_2_ and 95% air (v/v), and a spectrophotometer reader (Power-Wave XS microplate-reader, BioTek Instruments, Madrid, Spain) was used to register the absorbance of the soluble formazan product generated at 450 nm (reference 650 nm). 100% viability was set by control normoxic cells treated only with medium, and control cells were added 0.001–1% DMSO in every assay.

#### Assessment of LDH activity

To evaluate their anti-necrotic cell activity, SH-SY5Y cells were cultured in 48-well dishes (density of 1.25 × 10^5^ cells/well) and put under the OGD-R24h procedure (including ischemia and reperfusion controls), adding the testing compounds at 0.01–1000 µM. Media of every well were collected into properly labelled Eppendorf tubes and stored at − 20 °C until measurement (samples would be needed to measure extracellular NADH). Afterwards, each well was added a 0.1 M phosphate buffer with 0.5% Triton X-100, pH 7.5, and the attached cells were scratched from the bottom of the well. The obtained samples were subjected to a 13,000 rpm centrifugation to subsequently measure intracellular NADH generated by collecting the soluble fractions. Finally, LDH activity was quantified as the degree of absorbance declined at 340 nm, indicating the oxidation of NADH to NAD^+^ as described^10,16^. Results are expressed as the LDH activity of the extracellular medium referred to the total LDH activity after cell lysis.

#### Measurement of caspase-3 activity

For apoptosis assays, human neuroblastoma cells were seeded in 48-well culture plates (density 1.25 × 10^5^ cells/well approx.) and underwent the OGD procedure or were treated as controls, as described above. After said treatment, cells were incubated in the presence of the different nitrones and compounds at 0.01–1000 µM or used as positive controls of damage for 24 h (R24h). Thereafter, cells were lysed at 4 °C in a medium containing 20 mM ethylenediaminetetraacetic acid, 5 mM Tris/HCl (pH 8.0), and 0.5% Triton X-100, and centrifuged at 13,000 rpm for 10 min. Specific Caspase-3 activity expressed as arbitrary fluorescence units [(AFU)/µg protein/h], was assessed by making use of DEVD-AMC (66081; BD Biosciences PharMingen, Madrid, Spain), a fluorigenic substrate peptide, and by using the Bradford method to determine protein quantity, as described previously^10^.

#### Evaluation of ROS formation

In order to estimate the quantity of superoxide anion formation, SH-SY5Y cells were seeded in 48-well plates at a density of 1.25 × 10^5^ cells/well. Firstly, cultured cells were exposed to OGD treatment for 3–4 h or maintained in normoxic conditions (controls). Then, medium was replaced with oxygenated Dulbecco’s: Ham’s F12 medium with glucose, 1% Antibiotic–Antimycotic, 1% Gentamicin and 10% FCS. Cells were incubated in the absence (controls) or presence of indicated concentrations of the drugs of study, at 37 °C for 3 h. Later, dihydroethidium [DHE (HEt; Molecular Probes, ThermoFisher Scientific, Madrid, Spain)], a fluorigenic redox indicator that reacts with the superoxide anion,, was added to the medium and fluorescence was recorded every 15–30 s during a 15 min period, using excitation at 535 nm and emission at 635 nm in a spectrofluorimeter (Bio-Tek FL 600, BioTek Instruments, Madrid, Spain)^10^. For every condition, the linear regression of fluorescence data (expressed in arbitrary fluorescence units, AFU) was calculated, and the slopes (a) of the best fitting lines (y = ax) were considered as an index of superoxide production, as described previously^17^.

#### Estimation of lipophilicity as ClogP

Lipophilicity is an important physicochemical property related to biological efficacy and ADME properties. The logarithm of the partition coefficient between n-octanol and water (log Coctanol/Cwater), also known as ClogP value of a compound, measures the molecule’s hydrophilicity. Low hydrophilicities, or high lipophilicities, and thus, high ClogP values are correlated to low absorption or permeation. The Bioloom platform from Biobyte Corp was used for the theoretical calculation of lipophilicity as Clog P values (BioByte Home Page. Available online: http://www.biobyte.com).

### In vitro antioxidant activity and anti-inflammatory assays of **QNs 1–6**

Several assays should be used since factors such as solubility or steric hindrance can be varied and the antioxidant ability of a compound in a variety of milieus may be evaluated. All the assays were performed through a spectrophotometric measurement and a certain reaction time in order to obtain reproducible results. Three different in vitro protocols were used: the inhibition of lipid peroxidation (LP) induced by 2,2′-azobis(2-amidinopropane) dihydrochloride (AAPH) in the presence of atmospheric oxygen, the competition of the tested compounds with DMSO in terms of hydroxyl radical scavenging activity, and the 2,2′-azinobis-(3-ethylbenzothiazoline-6-sulfonic acid (ABTS)· decolorization assay. In vitro inhibition of soybean lipoxygenase (LOX) was used to determine anti-inflammatory activity. All these compounds were obtained from Aldrich Chemical Co. Milwaukee, WI, (USA). A Lambda 20 (Perkin–Elmer-PharmaSpec 1700) UV–Vis double beam spectrophotometer was used for all the assays.

#### Inhibition of linoleic acid peroxidation (ILPO)

Screening studies confirmed that the reaction with AAPH led to clean oxidation. Azo compounds like AAPH generate free radicals through spontaneous thermal decomposition. This makes them useful for free radical production studies in vitro. According to our previous publication^8^ 10 μL of a 16 mM linoleate sodium solution and 0.93 mL of a 0.05 M phosphate buffer (pH 7.4), prethermostatted at 37 °C, were added to the UV cuvette. Then, it was inserted the free radical initiator AAPH 50 μL of a 40 mM AAPH solution at 37 °C in the presence of air and 10 μL of the tested compounds diluted in DMSO. The oxidation of linoleic acid sodium salt results in a conjugated diene hydroperoxide. The absorbance was recorded at 234 nm. Trolox was used as a reference compound and positive control.

#### Competition of the tested compounds with DMSO for hydroxyl radicals

Hydroxyl radicals were generated by the Fe^3+^/ascorbic acid system and detected by the determination of formaldehyde generated by the oxidation of DMSO. EDTA (0.1 mM), Fe^3+^ (167 μM), DMSO (33 mM) in phosphate buffer (50 mM, pH 7.4), the tested compounds (100 µM) and ascorbic acid (10 mM) were mixed in test tubes, and incubated at 37 °C for 30 min according to our previously published method^8^. The reaction was stopped by adding CCl_3_CO_2_H (17% w/v), and the percentage (%) scavenging activity of the tested compounds for hydroxyl radicals was calculated. Trolox was used as a positive control.

#### ABTS^**·**+^-decolorization assay in ethanolic solution for antioxidant activity

ABTS was dissolved in water to a 7 mM concentration. ABTS radical cation (ABTS^•+^) was produced by reacting ABTS stock solution with 2.45 mM potassium persulfate and allowing the mixture to stand in the dark at room temperature for 12–16 h before use. For the present study, the ABTS^•+^ solution was diluted with absolute ethanol to an absorbance of 0.70 at 734 nm. Stock solutions (10 mM) of the tested compounds in DMSO were diluted so that, after introduction of a 10 µL aliquot of each dilution into the assay, an absorbance between 20 and 80% inhibition of the blank absorbance was observed. Ten milliliter (10 mL) aliquots of each sample were added into the ABTS^•+^ solution (990 µL) and the absorbance was recorded at room temperature whereas the absorbance of the samples 10 µL in ethanol (990 µL) was taken 1 min after the initial mixing. Trolox was used as a positive control. The assay was performed as previously described^8^. The absorbances of the mixed solution were recorded after 1 min at 734 nm.

Free radical scavenging activity was expressed as inhibition percentage and was calculated using the formula (A0–A1)/A0 × 100, where A0 was the control absorbance and A1 was the sample absorbance.

#### In vitro inhibition of soybean LOX

In vitro inhibition of soybean LOX was followed the previously described method^8^. The tested compounds were dissolved in DMSO as 10 mM stock solutions. 10 μL were incubated at room temperature with sodium linoleate (100 mM) and 0.2 mL of enzyme solution (1/9 × 10^−4^ w/v in saline) in buffer pH 9 (tris) at room temperature (final volume 1 mL). The conversion of sodium linoleate to 13-hydroperoxylinoleic acid at 234 nm was recorded. The results were compared with the appropriate standard inhibitor NDGA (IC_50_ = 0.45 μM). Several concentrations were used for the determination of IC50 values. The results are given in Table 1 expressed as IC_50_ values or % inhibition at 100 μM and compared with the appropriate standard inhibitor. Nordihydroguaiaretic acid (NDGA) (Aldrich Chemical Co. Milwaukee, WI, (USA), was used as a positive control. To determine the IC_50_ values, several dilutions of compounds were used. Blank determination served as a negative control.

### Infarct size evaluation in an in vivo* pMCAO* stroke model

To study of the effects of the selected compound on stroke recovery, administration of the vehicle and **QN6** was performed intraperitoneally, to 8 week old male C57BL/6J mice (Harlan) weighing 25–30 g. All procedures associated with animals experiments were carried out under a protocol approved by the Ethical Committee of the Spanish National Research Council (CSIC), (Madrid, Spain, PROEX 116.6/21, Certificate 10/081517.9/21) and were performed according to the ARRIVE guidelines (https://arriveguidelines.org). A special effort was made to keep to a minimum necessary the number of animals to achieve adequate significance. For surgery, anaesthesia induction was carried out with 3% isoflurane (in 70% NO_2_, 30% O_2_), followed by 2% isoflurane for maintenance during stroke procedure. Rectal temperature was maintained at 36.5 °C with the use of a heating pad. The common branch of the middle cerebral artery was, after craniotomy, exposed and occluded permanently by suture ligation as previously reported^18^. To ensure a complete artery occlusion during surgery, cortical blood flow was monitored by non-invasive laser Doppler flowmetry, as a quality control, with the aid of Perimed equipment (PeriFlux System 5000 Stroke Model Monitor, Perimed, Järfälla, Sweden). The study was exclusively performed in animals that showed post-ligature a drop of blood flow of at least 65%. Animals subjected to surgery for longer than 15 min were excluded of the study. Physiological parameters were maintained as previously reported^10^. Experiments were performed in each of the following groups: (a) sham operated (n = 6 animals); (b) pMCAO vehicle control group (saline buffer containing 29% DMSO) (n = 7), and (c) pMCAO QN6 treated group (85 mg/kg QN6 dissolved in vehicle) (n = 8). Drug administration was 15 min after arterial ligature.

MRI is the most commonly used diagnostic tool for stroke. Diffusion-weighted images (WI) were acquired in T2 (T2WI) as a tool to obtain precise images of the vascular edema, with a resolution limit of 0.8–1 mm^3^. This information is needed to delineate lesion boundaries for assessment of intracranial edema location and growth pattern evaluation. In this study, determination of infarct size in vehicle and **QN6** treated mice was performed in sequential MRI coronal 0.8–1 mm-thick brain slices as reported previously in our laboratory^18,19^. MRI studies were performed at BioImaC (ICTS BioImagen Complutense University), node of the ICTS ReDIB (https://www.redib.net/) using one tesla (1 T) benchtop MRI scanner ICON-1T; Bruker BioSpin (GmbH, Ettlingen, Germany). The system consists of a 1T permanent magnet (without extra cooling required for the magnet) with a gradient coil that provides a gradient strength of 450 mT/m. The animal monitoring systems and the solenoid mouse head RF are integrated into the bed and they allow the animals to be handled on with accurate positioning of the coil and full control of anaesthesia and body temperature. MRI experiment consisted of three dimensional T2WI used to evaluate the vascular edema. Three-dimensional T2WI were acquired using a rapid acquisition with relaxation enhancement (RARE) technique, with a repetition time (TR) = 2500 s, echo train length = 12, interecho interval = 18 ms (resulting in an effective echo time (TE) = 90 ms), number of average = 1, field of view (FOV) = 18 × 18 × 14 mm. The acquired matrix size was 120 × 120 × 28 (resolution 0.150 × 0.150 × 0.500 mm) and total acquisition time was ~ 12 min. All MRI data was analyzed using ImageJ software.

All methods described in the manuscript were carried out in accordance with relevant guidelines and regulations.

### Statistical analysis

Data obtained in cell cultures have been expressed as mean ± SEM of results from at least three independent experiments of different batches, each performed in triplicate. Statistical comparisons between the different experimental treatments in cells were performed by one-way analysis of variance (ANOVA), which was followed by the Holm-Sidak post-test when the analysis of the variance was significant. A p value < 0.05 was assumed to be statistically significant. The fitting curves for EC_50_ and maximal activity determinations were carried out according to SigmaPlot v.11 (Systat Software INC., 2012); https://systatsoftware.com/sigmaplot/download-sigmaplot-software/).

The curve adjustments to estimate the EC_50_ were carried out by non-linear ponderated regression analysis of minimal squared, using logistic curves f1 = min + (max − min)/(1 + (x/EC_50_)^(− Hillslope).

Each in vitro, cell-free experiment was performed at least in quadruplicates and the standard deviation of absorbance was less than 10% of the mean. Statistical comparisons were performed using Student’s *t* test.

For in vivo MRI study, statistical significance was evaluated by two-way ANOVA followed by post hoc Bonferroni’s test to compare paired experimental points (**p < 0.01).

### Ethical statement

All methods were carried out in accordance with relevant guidelines and regulations.

## Supplementary Information

Supplementary Figures.

## Abbreviations

AAPH: 2,2′-Azobis(2-amidinopropane) dihydrochloride
BBB: Blood–brain barrier
DHE: Dihydroethidium
DPPH: 1,1-Diphenyl-2-picrylhydrazyl radical
hBuChE: Human butyrylcholinesterase
hMAO-B: Human monoamine oxidase B
ILPO: Inhibition of lipid peroxidation
IR: Ischemia–reperfusion
LDH: Lactate dehydrogenase
LOX: Lipoxygenase
LP: Lipid peroxidation
MAA: Maximal antioxidant activity
MNA: Maximal neuroprotective activity
NAC: *N*-Acetyl-l-cysteine
NDGA: Nordihydroguaiaretic acid
OGD: Oxygen–glucose deprivation
OGR: Oxygen and glucose resupply
OS: Oxidative stress
PNB: α-Phenyl-*N*-tert-butylnitrone
QNs: Quinolylnitrones
ROS: Reactive oxygen species
SNP: Sodium nitroprusside
