# Supplementary material for: Neuroprotective and antioxidant properties of new quinolylnitrones in in vitro and in vivo cerebral ischemia models
Source: Sci Rep. 2023 Feb 17;13:2865. doi: 10.1038/s41598-023-29929-7 (PMC9938201; doi:10.1038/s41598-023-29929-7)
Supplement: Supplementary file 1 — Supplementary Figures. [file 41598_2023_29929_MOESM1_ESM.pdf]

Supplementary Material

Neuroprotective and Antioxidant Properties of New Quinolylnitrones in *in vitro* and i*n vivo* Cerebral Ischemia models

Beatriz Chamorro,[1,2] Sara Izquierdo-Bermejo,[1] Julia Serrano,[3] Dimitra Hadjipavlou-Litina,[4] Mourad Chioua,[5] Francisco López-Muñoz,[2,6] José Marco-Contelles,[5,7] Ricardo Martínez-Murillo,[3] and María Jesús Oset-Gasque,[1,8,*]

1 Department of Biochemistry and Molecular Biology II, Faculty of Pharmacy, Complutense University of Madrid, Plaza Ramón y Cajal s/n, Ciudad Universitaria, 28040 Madrid, Spain. B.C., beatrcha@ucm.es S.I-B., sizqui03@ucm.es; MJ.O-G., mjoset@ucm.es.

2 Faculty of Health, Camilo José Cela University, Villanueva de la Cañada, Madrid, Spain; B.C., beatrcha@ucm.es, F.L-M., flopez@ucjc.edu.

3 Neurovascular Research Group, Department of Translational Neurobiology, Cajal Institute (CSIC), Madrid, Spain J.S., jserrano@cajal.csic.es; R.M-M., r.martinez@cajal.csic.es.

4 Department of Pharmaceutical Chemistry, School of Pharmacy, Faculty of Health Sciences, Aristotle University of Thessaloniki, Thessaloniki 54124, Greece. D.H-L., hadjipav@pharm.auth.gr.

5 Laboratory of Medicinal Chemistry, Institute of Organic Chemistry (CSIC), Juan de la Cierva 3, 28006-Madrid, Spain. M.C., mchioua@gmail.com; J.M-C., jlmarco@iqog.csic.es.

6 Neuropsychopharmacology Unit, "Hospital 12 de Octubre" Research Institute, Madrid, Spain.

7 Center for Biomedical Network Research on Rare Diseases (CIBERER), CIBER, ISCIII, Madrid, Spain

8 Instituto de Investigación en Neuroquímica. Universidad Complutense de Madrid. Ciudad Universitaria, 28040 Madrid, Spain.

* Correspondence: María Jesús Oset-Gasque (MJ.O-G.) (mjoset@ucm.es), Tel.: +34-1-394-1788.

Content

Figures S1-S4……….………………………………………………………2S-3S

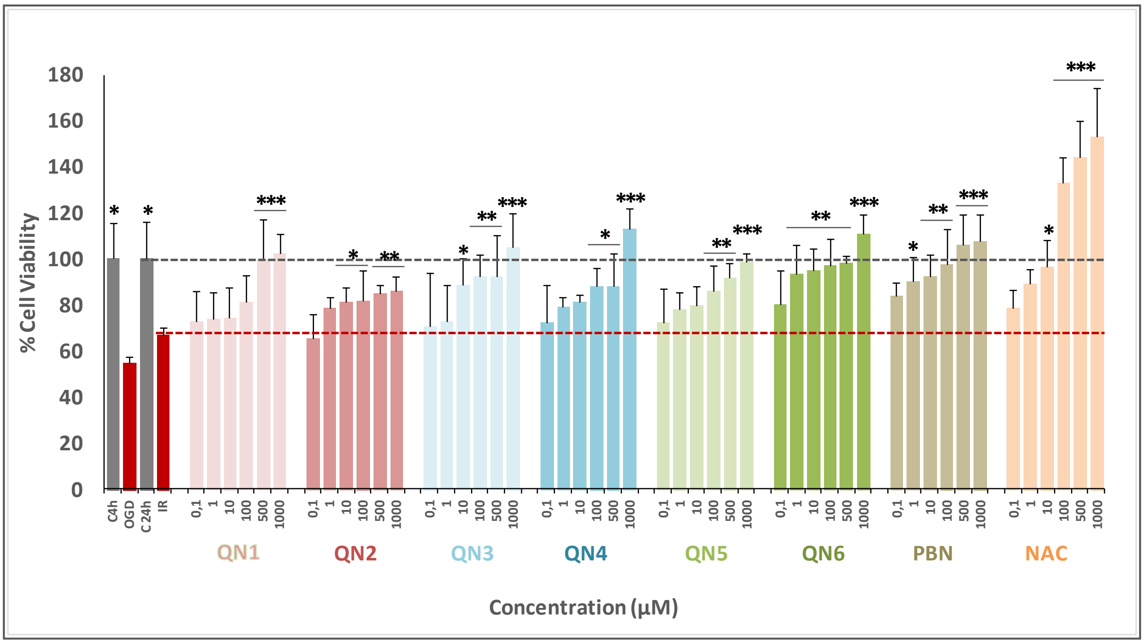

Figure S1. Neuroprotective effect of QNs 1-6, PBN and NAC on SH-SY5Y human neuroblastoma metabolic activity after oxygen glucose deprivation (4h) and ischemic reperfusion (24h) (IR). Bars show % cell viability at the indicated concentrations of **QNs 1-6, PBN** and **NAC**. Values are the mean ± SEM of three experiments, each one performed in triplicate. The statistics compares differences with IR condition alone (red dotted line) at *P <0.05, **P<0.01 and ***P< 0.001 (one-way ANOVA, followed by Holm–Sidak analysis as a test post hoc.

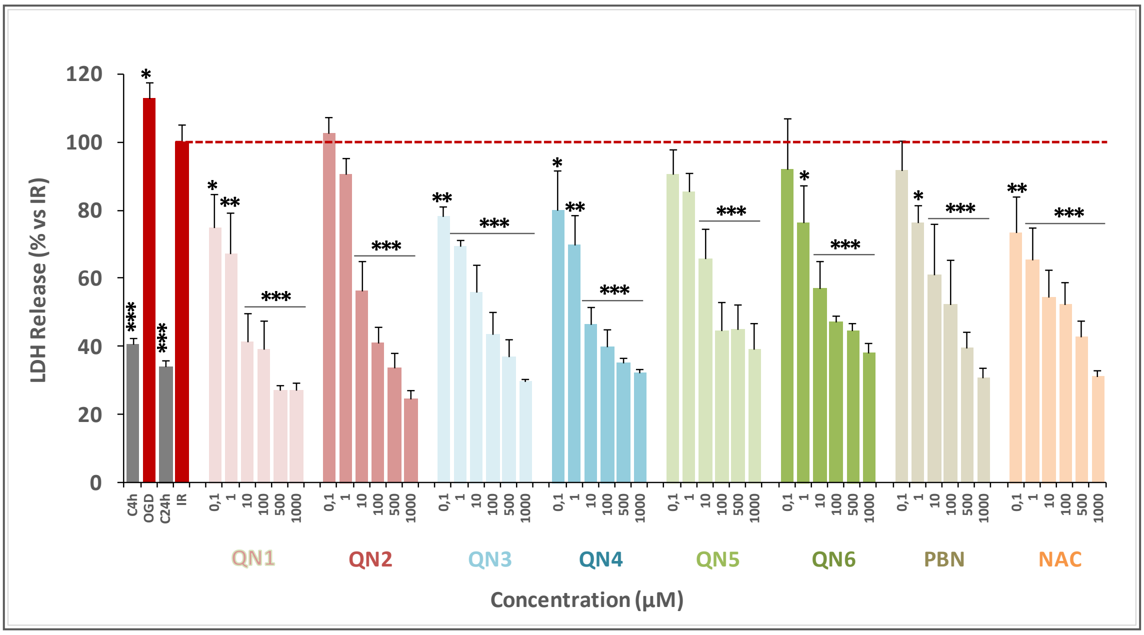

Figure S2. Effect of QNs 1-6, PBN and NAC on the lactate dehydrogenase release in SH-SY5Y cells after ischemia reperfusion. Bars show % LDH release after OGD (4 h) and IR (24 h), without treatment (IR 24 h) or treated with **QNs 1-6, PBN**, or **NAC**, at the indicated concentrations. Values are the mean± SEM of three experiments, each one performed in triplicate, and compared the effect of the different compounds after IR (24 h) (or controls) with IR (24 h) alone (red dotted line), i-e. in the absence of these compounds. Data were statistically analyzed by one-way ANOVA, followed by Holm–Sidak as test post hoc. * p < 0.05, ** p < 0.01, and *** p < 0.001.

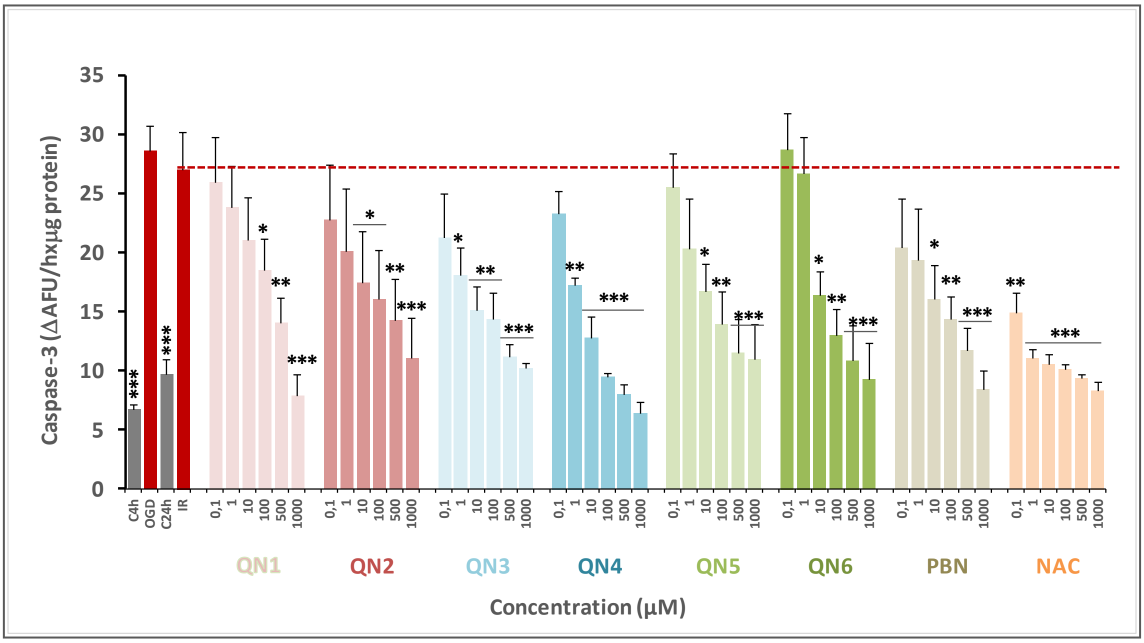

Figure S3. Neuroprotective effects of QNs 1-6, PBN and NAC against apoptotic cell death induced by IR treatment in SHSY5Y human neuroblastoma cells. Bars show caspase 3 activity (ΔAFU/min/μg protein) after OGD (4 h) and IR (24 h), without treatment (IR alone) or treated with nitrones **QNs 1-6**, **PBN**, and **NAC**, at the indicated concentrations. Values are the mean ± SEM of three experiments, each one performed in triplicate. The statistics compares the differences between caspase 3 activity for different compounds tested against IR condition alone at *$P < 0.05$, **$P < 0.01$ y ***$P < 0.001$ (ANOVA one way). AFU = arbitrary fluorescent units.

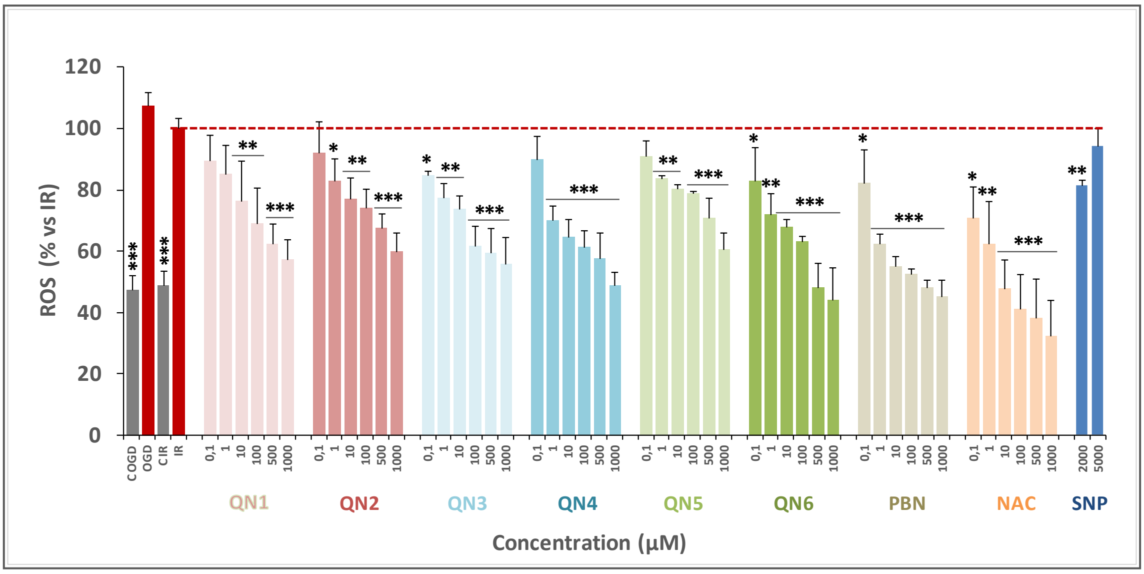

Figure S4. Inhibitory effects of QNs 1-6, PBN, and NAC on ROS (superoxide) production in SHSY5Y human neuroblastoma cell cultures exposed to OGD (4 h) and 3 h reperfusion (IR). Bars show the percentage of ROS formed after OGD and IR, with or without nitrones **QN 1-6** or **PBN** and **NAC**, at the indicated concentrations. Values are mean ± SEM of three experiments, each one performed in triplicate. Values for ROS in basal conditions (Control IR) were calculated as 0,135 ± 0,023 (mean ± SEM) AFU/min/100.000 cells (n = 6). The statistics compares the effect of IR against controls or the effect of the different compounds respect to IR condition alone at *$p < 0.05$, **$p < 0.01$, ***$p < 0.001$ (one-way ANOVA followed by Holm–Sidak analysis post hoc).
